# TAZ upregulates MIR‐224 to inhibit oxidative stress response in multiple myeloma

**DOI:** 10.1002/cnr2.1879

**Published:** 2023-08-04

**Authors:** Samuel O. Abegunde, Stacy Grieve, Tony Reiman

**Affiliations:** ^1^ Department of Biology University of New Brunswick Saint John New Brunswick Canada; ^2^ Dalhousie Medicine NB Saint John New Brunswick Canada; ^3^ Cytel Inc Toronto Ontario Canada; ^4^ Saint John Regional Hospital Saint John New Brunswick Canada

**Keywords:** miR‐224, multiple myeloma, NRF2, oxidative stress, TAZ

## Abstract

**Background:**

Oxidative stress within the bone marrow niche of multiple myeloma contributes to disease progression and drug resistance. Recent studies have associated the Hippo pathway with miRNA biogenesis and oxidative stress in solid tumors. Oxidative stress and miRNA pathway inter‐relates in several cancers. Our group recently showed that TAZ functions as a tumor suppressor in MM. However, the role of TAZ in oxidative stress in MM is unknown.

**Aims:**

We sought to examine the role of TAZ in myeloma cells' response to BM oxidative stress. We postulated that TAZ might be associated with an oxidative stress phenotype and distinct miRNA signature in MM.

**Methods and Results:**

Using human myeloma cell lines and clinical samples, we demonstrate that TAZ promotes myeloma cells' sensitivity to oxidative stress and anticancer‐induced cytotoxicity by inducing miR‐224 to repress the NRF2 antioxidant program in MM. We show that low expression of TAZ protein confers an oxidative stress‐resistant phenotype in MM. Furthermore, we provide evidence that overexpression of miR‐224 in myeloma cells expressing low amounts of TAZ protein inhibits cell growth and enhances sensitivity to anti‐myeloma therapeutics.

**Conclusion:**

Our findings uncover a potential role for TAZ in oxidative stress response in MM via the miR‐224‐NRF2 molecular pathway. This provides the scientific ground to explore miR‐224 as a potential molecular target to modify TAZ expression and enhance myeloma sensitivity to treatment.

## 1 INTRODUCTION

Multiple myeloma (MM) develops when normal plasma cells undergo malignant changes and accumulates within the bone marrow (BM).^1^, ^2^, ^3^ These malignant plasma cells secrete excessive amounts of abnormal monoclonal immunoglobulins.^1^, ^2^, ^3^ Immunoglobulin synthesis by plasma cells generates excess free radicals and oxidative stress that may act as potential growth‐promoting or inhibitory signals in a context‐dependent manner.^4^ In this context, recent studies suggest that the oxidative stress within the BM stroma of MM patients contributes to tumourigenesis and anticancer resistance.^5^, ^6^, ^7^ Indeed, as normal plasma cells (NPC) transform into myeloma cells, they undergo several genetic and epigenetic changes that reshape the antioxidant response mechanisms.^5^ Studies in solid tumors suggest that the Hippo signaling pathway (HP) is involved in oxidative stress and orchestrates tumourigenesis through several direct or indirect mechanisms.^8^, ^9^, ^10^, ^11^ The HP consists of two upstream serine–threonine kinase pairs, including MST1/MST2 and LATS1/LATS2. Downstream of the HP are two WW‐domain‐containing proteins: Yes‐associated protein (YAP) and transcriptional coactivator with PDZ‐binding motif (TAZ) that mediates its output.^8^, ^9^, ^10^, ^11^ Both proteins are transcriptional coactivators that bind to other transcription factor partners, such as the TEAD protein family (TEAD) to induce the expression of their targets.^8^, ^9^, ^10^, ^11^ We and others recently reported that YAP/TAZ act as tumor suppressors in MM.^12^, ^13^, ^14^ However, whether TAZ mediates oxidative stress response in MM is still unknown. YAP/TAZ may regulate global miRNA biogenesis.^15^, ^16^, ^17^ Recent reports suggest that miRNA and oxidative stress programs are interconnected in many diseases, including cancer.^18^, ^19^ In line with these findings, several studies have shown dysregulated miRNA expression in myeloma cells.^20^, ^21^, ^22^, ^23^ Despite these reports, the molecular mechanisms underlying oxidative stress response in MM are still poorly defined; no study has examined the relationship between TAZ and miRNA or oxidative stress in MM.

NRF2 is a key transcriptional regulator of the oxidative stress response in some solid tumors and hematological cancer.^24^, ^25^, ^26^ Therefore, we postulated that TAZ might act directly or indirectly through specific miRNAs to modulate oxidative stress response in myeloma cells. Using a panel of human myeloma cell lines (HMCLs) and publicly available patient data sets, we showed that TAZ upregulates miR224 expression to repress NRF2 antioxidant mechanisms in myeloma cells. We further show that low TAZ or miR‐224 expression is associated with oxidative stress‐resistant phenotype in MM. Finally, we provide evidence that upregulating miR‐224 expression may increase the susceptibility of myeloma cells to antimyeloma agents.

## 2 MATERIALS AND METHODS

### 2.1 Cell culture assays

Human myeloma cell lines, including high‐TAZ expressing cells (DELTA47 and MOLP8) and low ‐TAZ expressing HMCLs (KMS27 and KHM1B) initially identified by us^13^, ^14^ were cultured as previously reported.^14^ Where necessary, the cultured cells were also treated with hydrogen peroxide (H_2_O_2_) at different concentrations to induce oxidative stress. Lenalidomide (LMD) and bortezomib (BTZ) were purchased from Cayman Chemicals.

### 2.2 Lentiviral production, target cell transduction, and puromycin selection

DELTA47 cells were transduced by standard spinoculation technique using lentivirus‐expressing sgTAZ or pLenti CRISPR plasmid DNA to generate knockout TAZ or pLenti myeloma cell models respectively. For the viral transduction, DELTA47 cells (1 × 10^5^) were seeded in 6‐well plates with ~80% confluency, lentiviral supernatants were filtered and added in a dropwise fashion to the cells in the presence of polybrene. Mixtures were incubated at room temperature for 20 min and centrifuged at 800 *g* for half an hour. Puromycin selection was performed using a concentration of 1.0 μg/mL based on the killing curves. Western blot technique was used to verify the knockout of TAZ in the propagated surviving cells. We further examined whether the effect of TAZ on the expression of miR‐224 is mediated through TEAD by using lentiviral transduction to generate KMS27 or KHM1B cells that express TAZ, or a TEAD‐binding mutant of TAZ (TAZ‐F52A/F53A) or empty vector control (WPI) as previously described.^13^ The CRISPR knockout and WPI overexpression plasmid systems were a kind gift from Dr. Xiaolong Yang.

### 2.3 Transfection with synthetic microRNA

We transfected HMCLs, including KHM1B, KMS27, MOLP8, and DELTA47 with hsa‐miR‐224 mimic or inhibitor (ABM, British Columbia, Canada) following the Lipofectamine RNAiMAX protocol from Invitrogen. Briefly, myeloma cells seeded at a density of 2 × 10^5^ cells/well in 6‐well plates were cultured until 70%–80% confluent. The culture media were removed and replaced with a mixture of diluted miRNA mimics or inhibitors and Lipofectamine RNAiMAX Reagent in Opti‐MEM medium and incubated at 37°C for 24–48 h and analyzed. We used 10 nM of miRNA mimic or inhibitor, to give a final concentration of 25 pmol per well of a 6‐well plate.

### 2.4 Cell‐viability assays

Following transfection, cells were plated in triplicate at a density of 1 × 10^4^ per well in 24‐well plates for 72–96 h, and Trypan blue exclusion assay was used to estimate the total number of viable and dead cells.

### 2.5 Growth inhibition assays

We plated 1 × 10^4^ cells in triplicates in 96‐well plates for 48 h, followed by drug treatment with LMD or BTZ for 48–72 h, and measured their viability using the Presto Blue assay (Thermo Fisher). We used Trypan blue exclusion assay to measure the viability of cells in H_2_O_2_ treatment assays.

### 2.6 Cellular ROS detection

Intracellular ROS generation was determined using the Dichloro‐dihydro‐fluorescein diacetate (DCFDA) assay kit (Cayman Chemical, USA) according to the manufacturer's instructions. Briefly, DELTA47‐pLENTI and KO‐TAZ DELTA cells were seeded at a density of 1 × 10^4^ cells/well in V‐bottom 96‐well plates and treated with a variable amount of H_2_O_2_ for 1 h, while N‐acetyl‐l‐cysteine (NAC) was used as a negative control. To detect ROS, cells were stained using an oxidation‐sensitive dye DCFDA at a final concentration of 10 μM, incubated for 30 min in the dark, and immediately assessed on the fluorescence plate reader at an excitation wavelength of 480 nm and emission wavelength of 510 nm. The fluorescent peak in H_2_O_2_‐treated cells was compared to the peaks in controls (NAC pretreated cells and untreated cells). Triplicate wells were used for all conditions.

### 2.7 Cellular antioxidant production

Intracellular glutathione was determined using the GSH/GSSG ratio detection assay kit (Cayman Chemical, USA), according to the manufacturer's instructions. The amount of fluorescence signal was measured on the BioTek Synergy H4 Hybrid Microplate Reader at 490/520 nm.

### 2.8 Western blotting and antibodies

Protein lysates were obtained from cells using RIPA lysis buffer and measured using the Pierce BCA Protein Assay kit (ThermoScientific). Protein samples were separated by gel electrophoresis, incubated with the HRP Anti‐Rabbit IgG secondary antibody (1 in 10 000), and detected by chemiluminescence as previously described.^14^ Primary antibodies used include TAZ (1:1000), Caspase 3 (1:5000), and cleaved caspase 3 (1:500) (Abcam); NRF2 (1:1000) (ThermoFisher Scientific), GABRE (1:1000) (ThermoFisher Scientific), and β‐actin (1:5000) (Abcam) as control.

### 2.9 RNA and miRNA isolation and quantitative PCR

We extracted RNA from cells using the RNeasy kit (Qiagen), synthesized cDNA from 1 μg of RNA, and performed quantitative polymerase chain reaction (qPCR) using the SYBR Green technique as previously described.^14^ For the miRNA expression analysis, we used the mirVana miRNA isolation kit (Life Technologies) to extract total RNA enriched for miRNAs and measured RNA concentrations and quality on the Bioanalyzer. Complementary DNA was synthesized from total RNA using a miRNA cDNA synthesis kit (OriGene Technologies, Rockville, MD). miRNA expression was determined using SYBR Green qPCR microRNA detection assay (OriGene Technologies, Rockville, MD) with U6 primers as a reference control. All experiments were performed in triplicates. miRNA primer pairs are listed in Supplementary Table 1.

### 2.10 Computational microRNA target prediction and validation

We used TargetScan^27^, ^28^ and miRDB^29^ databases to identify potential miR‐224 target genes.

### 2.11 miRNA expression profiling

Total RNA was obtained from myeloma cells as previously described.^14^ MiRNA sequencing was done at Genome Quebec. Briefly, NEB small RNA library (bead size selection) was prepared, followed by miRNA sequencing analysis using NovaSeq6000 S4 PE100 sequencing lanes at a sequencing fraction of 0.0178 per sample. The miRNA seq data were analyzed by the Bioinformatic facility at Montreal Clinical Research Institute, Quebec.

### 2.12 Gene expression profiling using public datasets

The Gene Expression Omnibus^30^ and the Expression Atlas databases^31^ were interrogated for the expressions of TAZ and miR‐224 in the clinical samples of myeloma patients using GSE6477 data at probe sets 202132_s_at (TAZ) and 204537_s_at (miR‐224).

### 2.13 Statistical analysis

The GraphPad Prism V5 software was used to statistically analyze the data. We used the Student's *t*‐test to evaluate the mean difference between two groups, and ANOVA test for more than two groups. Data were correlated by simple linear regression method.

## 3 RESULTS

### 3.1 TAZ influences the sensitivity of myeloma cells to oxidative stress

**FIGURE 1:**
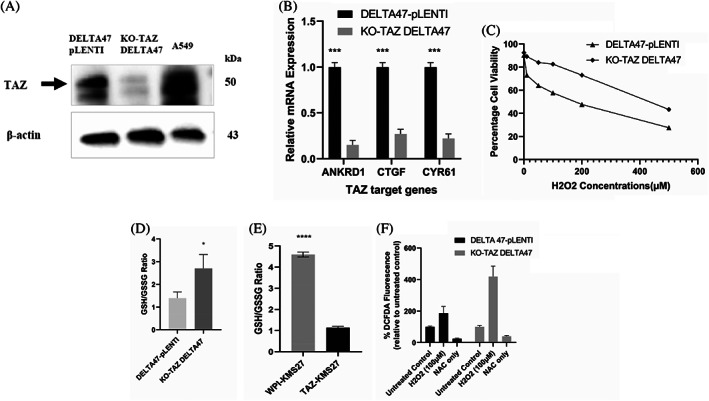
TAZ represses the oxidative stress‐resistant phenotype in MM. (A) Immunoblot of DELTA47 cells following CRISPR‐mediated TAZ knockout, β‐actin was used as a loading control. (B) Relative mRNA expression of selected TAZ target genes (ANKRD1, CTGF, and CYR61). rRNA as the reference gene. (C) Cell viability of KO‐TAZ DELTA47 compared to DELTA47‐pLENTI following exposure to varying concentrations of H_2_O_2_ in the cell culture system. Assessment of GSH/GSSG ratio in (D) KO‐TAZ and DELTA47‐pLENTI cells. (E) TAZ‐KMS27 and WPI‐KMS27 cells using a luminescence‐based assay. (F) Assessment of ROS generation using DCFDA assay in KO‐TAZ and DELTA47‐pLENTI cells.

We have previously characterized HMCLs into two groups based on TAZ expression status.^13^, ^14^ To investigate whether TAZ is involved in oxidative stress, we knocked out TAZ in a high‐TAZ expressing cell line (DELTA47) and evaluated the consequence of TAZ loss on oxidative stress response at varying H_2_O_2_ concentrations in cell culture. TAZ knockout resulted in the loss of TAZ protein (Figure 1A) and downregulation of TAZ target genes in DELTA47 cells (Figure 1B). A dose‐dependent decrease in cell viability was seen in both genotypes. TAZ knockout (KO‐TAZ) DELTA47 cells had a cell viability of 73.1 ± 5.2% at 100–200 μm H_2_O_2_ compared to 44.7 ± 3.7% for DELTA47 cells transduced with pLENTI‐CRISPR, suggesting an increased resistance to H_2_O_2_‐induced oxidative stress (Figure 1C). To further decipher the function of TAZ in oxidative stress response in MM, we performed a simple qualitative test by examining the ability of KO‐TAZ and DELTA47‐pLENTI myeloma cells to break down H_2_O_2_ to oxygen and water. KO‐TAZ and DELTA47‐pLENTI cells were treated with H_2_O_2_ (100 μM), and oxygen bubbles released were observed and compared subjectively. We observed that KO‐TAZ DELTA47 cells displayed a greater capacity to decompose H_2_O_2_ compared to DELTA47‐pLENTI cells (Supplementary Figure 1), suggesting high antioxidant ability. To further assess cellular antioxidative capacity in our TAZ knockout and TAZ expressing models, we examined the basal and induced levels of known antioxidant markers, including reduced glutathione (GSH) and GSH/oxidized glutathione (GSSG) ratio. Interestingly, KO‐TAZ DELTA47 displayed an increased GSH/GSSG ratio compared to DELTA47‐pLENTI cells (Figure 1D); in contrast, TAZ overexpressing KMS27 cells displayed a reduced GSH/GSSG ratio compared to WPI‐KMS27 (Figure 1E). Together, these findings suggest that TAZ suppresses antioxidant activity in MM, therefore, low TAZ expression may be associated with oxidative stress‐resistant phenotype in MM. We further sought to determine whether TAZ affects intracellular ROS levels in MM by using the oxidation‐sensitive probe DCFDA whose fluorescent signal intensity is directly proportional to the generation of ROS. We observed a more than 2‐fold increase in DCFDA fluorescent signals in KO‐TAZ DELTA47 cells at 100 μM compared to DELTA47‐pLENTI cells, suggesting a higher ROS generation in the KO‐TAZ model (Figure 1F).

### 3.2 TAZ upregulates miR‐224 in HMCLs

**FIGURE 2:**
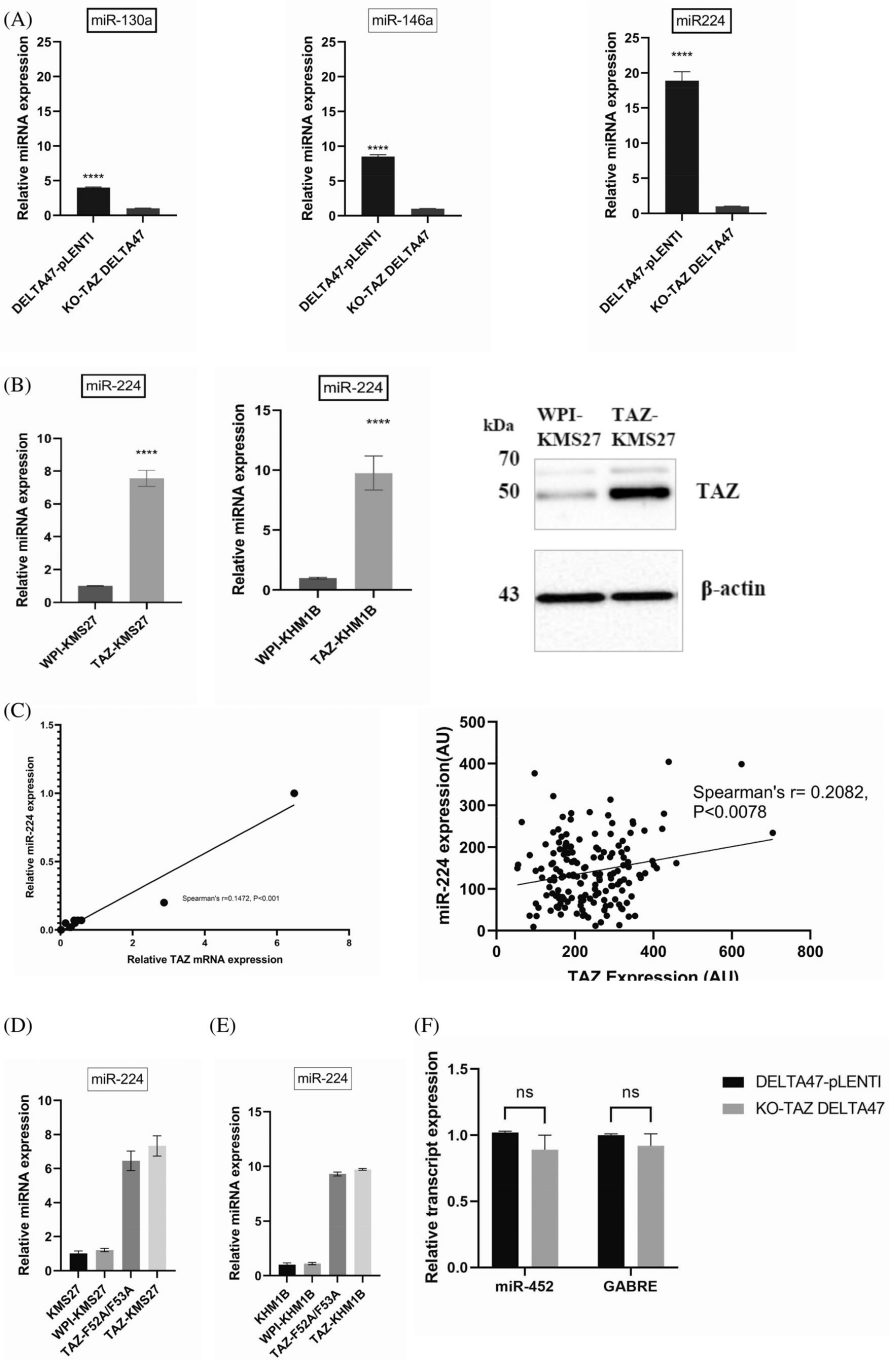
TAZ induces miR‐224 in HMCLs. (A) Expression of miR‐130a, miR‐146a and miR‐224 in KO‐TAZ and DELTA47‐PLENTI cells. (B) TAZ induces miR‐224 expression. miR‐224 expression in KMS27 and KHM1B stable cells overexpressing TAZ was assessed by qRT‐PCR 72 h post‐transduction. (C) TAZ expression positively correlates with miR‐224 expression in HMCLs (*n* = 12) and MM patients (*n* = 162) using GSE6477 data at probe sets 202132_s_at (TAZ) and 204537_s_at (miR‐224). Quantitative RT‐PCR analysis of miR‐224 in (D) KMS27 and (E) KHM1B cells expressing WPI, TAZ, or TAZ‐F52A/F53A. TEAD binding mutant of TAZ could not abort TAZ‐mediated upregulation of miR‐224. Experiments were performed in triplicate (*n* = 3, mean ± SD, ***p < .001). (F) miR‐452 and GABRE expression in the KO‐TAZ and DELTA47‐pLENTI myeloma cells.

Given the recently published articles showing that miRNA deregulation may be involved in oxidative stress response,^16^, ^17^, ^18^, ^19^ we conducted miRNA sequencing in the KO‐TAZ and DELTA47‐pLENTI myeloma cells to identify potential miRNAs which may be affected by TAZ. We cross‐compared the change in miRNA expression in the KO‐TAZ and DELTA47‐pLENTI myeloma cells. A total of 10 miRNAs were differentially expressed at a log2‐fold (FC > 2, adjusted *p* < .05) in KO‐TAZ DELTA47 cells compared with DELTA47‐pLENTI cells. Using qPCR, we validated three miRNAs, including miR‐130a, miR‐146a, and miR‐224 to be increased with TAZ expression in MM (Figure 2A). Given that the most dramatic change in the TAZ knockout model was seen with miR‐224, we focused on miR‐224. Consequently, we restored TAZ expression in low‐TAZ‐expressing cells, using lentiviral transduction as previously described,^13^ and investigate its function. Re‐expression of TAZ in KMS27 and KHM1B caused high expression of miR‐224 (Figure 2B). Furthermore, we performed pairwise Spearman correlation between miR‐224 and TAZ expression in 12 HMCLs and MM patient samples in publicly available databases to further evaluate their relationship. As evident in Figure 2C, TAZ expression positively correlates with miR‐224 expression in both HMCLs (*n* = 12, *p* < .001) and MM patient samples (*n* = 162, *p* < .0078). These findings suggest that TAZ upregulates miR‐224 in MM.

The TEAD proteins are the most implicated transcription factors that bind TAZ and mediate several TAZ functions.^32^ In KMS27 or KHM1B cells transduced with TEAD‐binding mutant of TAZ (TAZ‐F52A/F53A) or empty vector (WPI), we observed that mutation of TEAD binding sites on TAZ does not affect TAZ‐induced expression of miR‐224 (Figure 2D,E), suggesting that TAZ‐induced expression of miR‐224 probably occurs through a TEAD‐independent mechanism.

MiR‐224 is known to be located alongside miR‐452 on the gamma‐aminobutyric acid type A receptor epsilon subunit (GABRE) gene.^33^ Therefore, we shifted our attention to GABRE to determine its potential relationship with TAZ,^33^ by examining the expression of miR‐452 and GABRE in the KO‐TAZ and DELTA47‐pLENTI myeloma cells. There was no difference in the mRNA expression of GABRE in KO‐TAZ compared to DELTA47‐pLENTI myeloma cells (*p* > .05) (Figure 2F), suggesting that loss of TAZ does not affect the transcriptional expression of GABRE. Knockdown of GABRE using siRNA in DELTA47 cells caused only a slight decrease in the expression of miR‐224 (Supplementary Figure 4). These findings suggest that although TAZ‐upregulation of miR‐224 expression occurs independently of the GABRE, however, GABRE may partly contribute to miR‐224 expression in MM.

### 3.3 miR‐224 overexpression inhibits the capacity of myeloma cells to withstand oxidative stress injury

**FIGURE 3:**
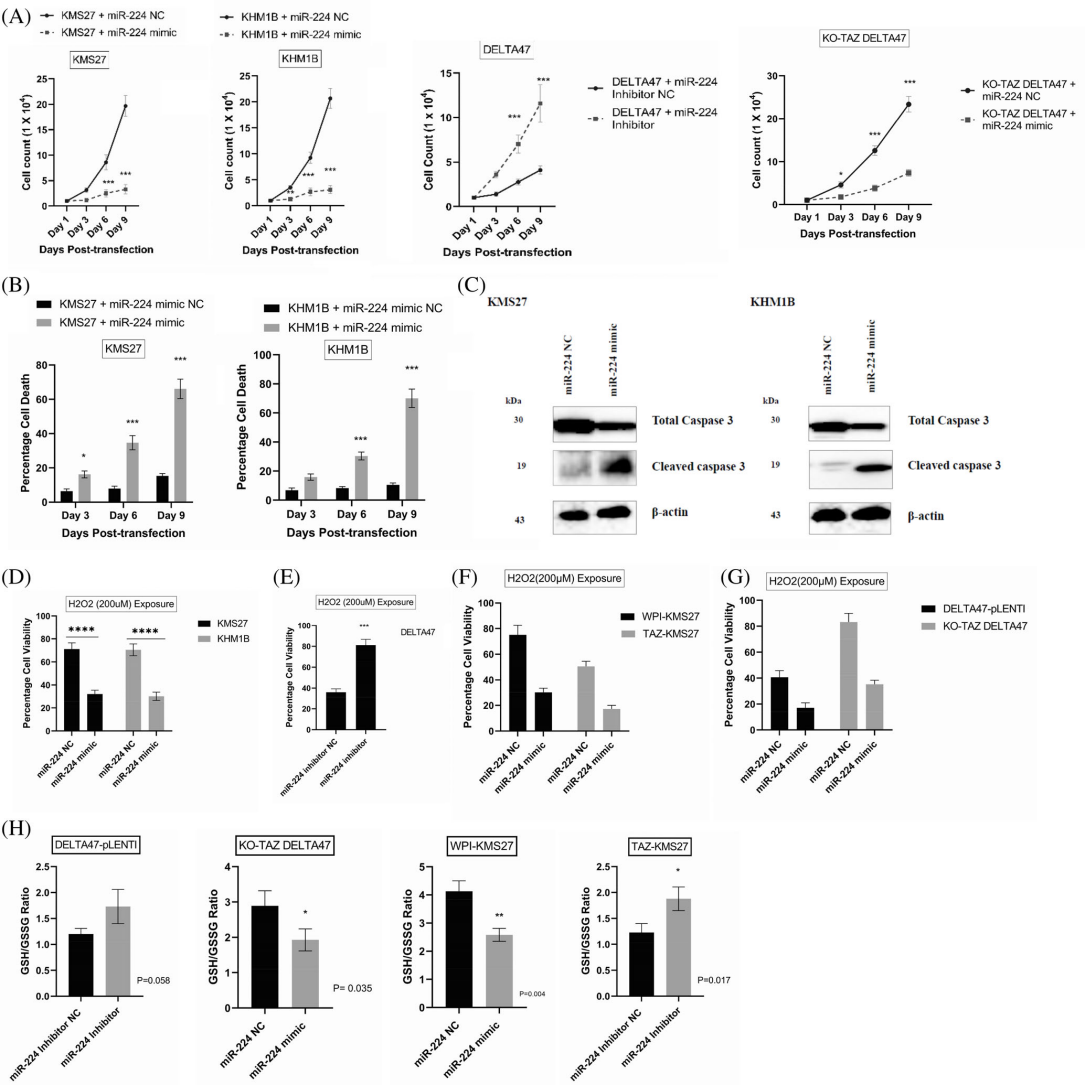
Overexpression miR‐224 inhibits tumor growth and enhances the susceptibility of HMCLs to oxidative stress. (A) Proliferation assay of KMS27, KHM1B, KO‐TAZ DELTA47, and DELTA47 cells transfected with miR‐224 mimics or inhibitors. Data are mean ± standard deviation (SD) of triplicates. *p ≤ .05 or ***p < .001 of miR‐224 mimic or inhibitor transfected cells compared with negative controls (NC), using ANOVA test. (B) Cell‐viability assay of selected cell lines described in panel (A) after 3‐, 6‐, or 9‐days post‐transfection. The percentage of cell death of miR‐224‐mimic transfected KMS27 and KHM1B cells increased significantly, compared with control cells. Data are mean ± SD of triplicates. *p ≤ .05 or ***p < .001 of miR‐224 mimic transfected cells compared with negative controls (NC), using the Student *t*‐test. (C) Immunoblot of KMS27 and KHM1B transfected with miR‐224 mimics or NC showing cleaved caspase3 expressions. β‐actin was used as a loading control. (D) H_2_O_2_‐induced oxidative stress assay. KMS27 and KHM1B were transfected with miR‐224 mimic or negative control followed by treatment with or without 200 μM of H_2_O_2_ for an hour and percentage cell viability relative to untreated control cells was assessed by Presto Blue assay. (E) DELTA47 cells were transfected with miR‐224 inhibitor or negative control followed by treatment with or without 200 μM of H_2_O_2_ for an hour and percentage cell viability was assessed by Presto Blue assay. (F) KMS27 cells overexpressing TAZ or WPI were transfected with miR‐224 mimic or negative control followed by treatment with or without 200 μM of H_2_O_2_ for an hour and percentage cell viability was assessed by Presto Blue assay. (G) KO‐TAZ DELTA47 or DELTA47‐pLENTI cells were transfected with miR‐224 mimic or negative control followed by treatment with or without 200 μM of H_2_O_2_ for an hour and percentage cell viability was assessed by Presto Blue assay. (H) Assessment of oxidative stress response using GSH/GSSG ratio in WPI‐KMS27 and KO‐TAZ DELTA47 transfected with miR‐224 mimic versus TAZ‐KMS27 and DELTA47‐pLENTI transfected with miR‐224 inhibitor.

Because of the positive correlation between miR‐224 and TAZ in HMCLs, we hypothesized that miR‐224 mediates tumor suppressor function in MM. Therefore, we transfected KMS27 and KHM1B with miR‐224 mimic oligonucleotides and examined their effect on proliferation and cell death. Over‐expression of miR‐224 in KMS27 and KHM1B cells resulted in a greater than three‐fold reduction in cell proliferation by day‐6 and day‐9 of transfection compared to NC‐transfected cells (Figure 3A). Similarly, overexpression of miR‐224 in KO‐TAZ DELTA47 significantly reduced cell proliferation from day‐3 to day‐9 (Figure 3A). In contrast, transfection of a miR‐224 inhibitor in DELTA47 cells increased their proliferative ability with a greater than 3‐fold increase on day‐6 and day‐9 (Figure 3A). Considering that cells expressing the miR‐224 mimic displayed reduced proliferation, we predicted this might be due to heightened cell death. As expected, overexpression of the miR‐224 mimic in KMS27 and KHM1B increased cell death in the cell viability assays. In KMS27 and KHM1B, cell death was observed from the third‐day post‐transduction and increased up to the ninth day (Figure 3B). We also demonstrated that miR‐224 overexpression triggers a proapoptotic response by reducing protein expression of total caspase 3 while increasing cleaved caspase 3 protein in KMS27 and KHM1B cells (Figure 3C).

Next, we sought to determine the role of miR‐224 in cellular response to oxidative stress by exposing miR‐224 myeloma cell models to equal concentrations of H_2_O_2_. Overexpression of miR‐224 in KMS27 and KHM1B decreased their percentage cell viability relative to NC‐transfected cells in H_2_O_2_ assays (Figure 3D), whereas overexpression of miR‐224 inhibitor in DELTA47 cells increased their percentage cell viability relative to their NC‐transfected counterparts in H_2_O_2_ assays (Figure 3E). These findings suggest that miR‐224 represses the resistance of myeloma cells to oxidative stress.

Furthermore, in TAZ‐KMS27 and WPI‐expressing KMS27 cells that were transfected with miR‐224 mimic, we observed a higher reduction in percentage cell viability in response to 200 μM H_2_O_2_ compared to cells transfected with NC miRNA. Specifically, percentage cell viability was further reduced from 50.5 ± 4.07% to 17.3 ± 2.71% in TAZ‐KMS27 following miR‐224 mimic transfection, while cell viability was also reduced from 75.1 ± 7.47 to 30.2 ± 3.35 in WPI‐KMS27 cells (Figure 3F). Similarly, KO‐TAZ DELTA47 and DELTA47‐pLENTI cells transfected with miR‐224 mimic also displayed lower cell viability relative to their NC transfected counterparts (Figure 3G). Transfection of miR‐224 mimic in WPI‐KMS27 and KO‐TAZ DELTA47 significantly reduced GSH/GSSG ratio these cells, whereas GSH/GSSG ratio was increased in both TAZ‐KMS27 and DELTA47‐pLENTI transfected with miR‐224 inhibitor (Figure 3H). These findings indicates that miR‐224 has an inhibitory effect on antioxidant capacity and can modulate TAZ function and oxidative stress response in MM.

### 3.4 miR‐224 downregulates NRF2 to modulate HMCLs' response to oxidative stress

**FIGURE 4:**
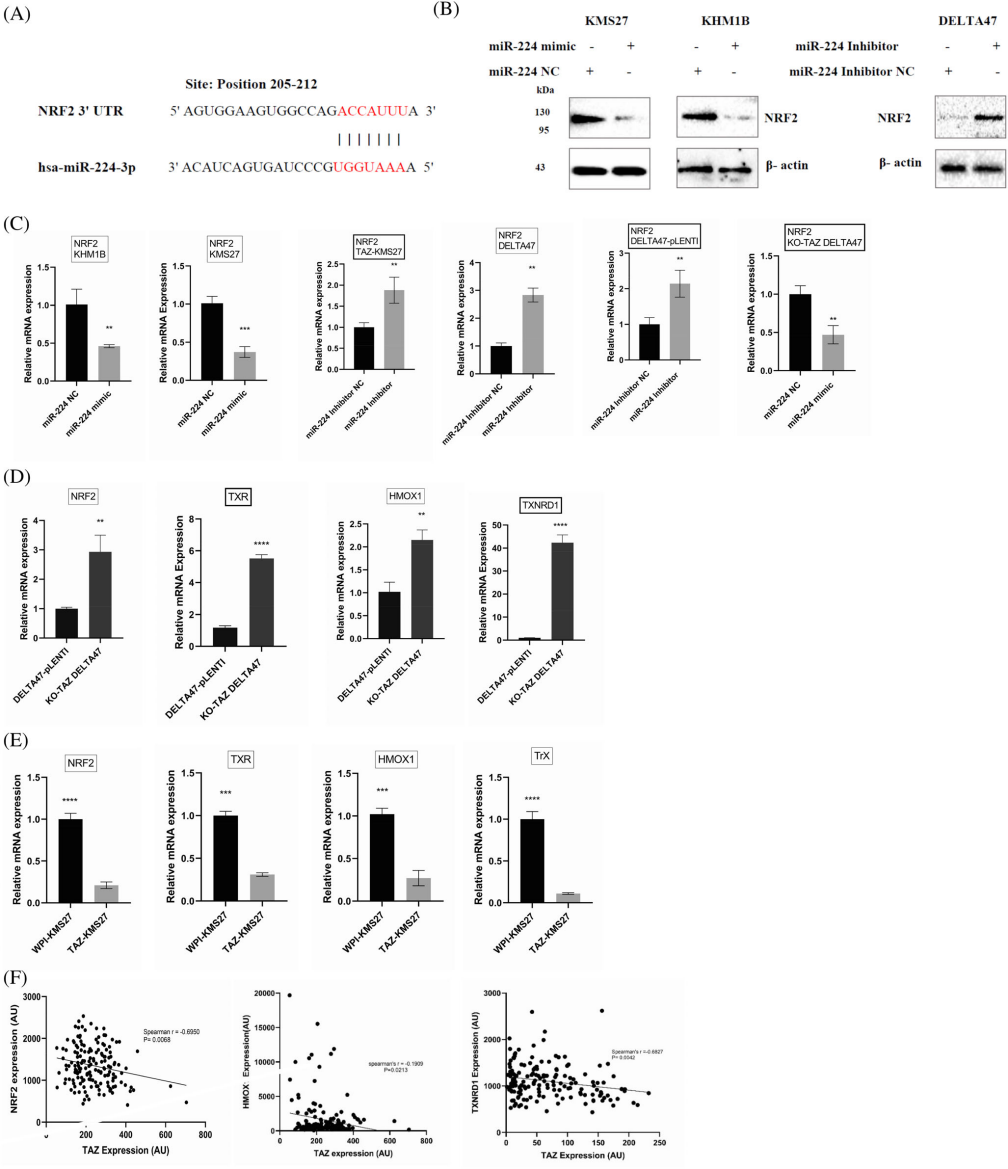
NRF2 is a target of miR‐224 regulated by TAZ. (A) Schematic diagram indicating the matched sequences between the 3′‐UTRs of NRF2, and the seed sequence of miR‐224. (B) miR‐224 represses NRF2 protein level. KMS27 and KHM1B cells were transfected with NC or miR‐224 mimic; DELTA47 cells were transfected with miR224 inhibitor. Cell lysates were subjected to immunoblotting. miR‐224 inhibition increases NRF2 protein level. (C) NRF2 mRNA expression in KMS27, KHM1B, and KO‐TAZ DELTA47 cells transfected with miR‐224 mimic, and DELTA47, DELTA47‐pLENTI and TAZ‐KMS27 cells transfected with miR‐224 inhibitor. (D) Basal mRNA expression of selected antioxidant genes (TRX, TXR, and NRF2) in DELTA47‐pLENTI compared to KO‐TAZ cells. (E) WPI‐KMS27 compared to TAZ overexpressing KMS27. Data are shown as mean ± standard deviation (SD) of triplicate experiments. **p < .01 compared with control, ***p < .001 compared with control; ****p < .00001 compared with control. (F) Expression of TAZ and antioxidant genes (NRF2, TXNRD1, and HMOX1) are anti‐correlated in MM patient samples combining data from GSE6477 using probe sets 202132_at (TAZ) and 208864_s_at (TXNRD1), 203665_at (HMOX1), and 201146_at (NRF2).

To decipher the underlying mechanism through which miR‐224 mediates myeloma response to oxidative stress, we used the TargetScan^27^, ^28^ and miRDB^29^ algorithms to predict potential miR‐224 targets. We observed that the 3′‐untranslated regions (UTRs) of NRF2 contained sequences complementary to the miR‐224 seed sequence (Figure 4A). We choose NRF2 due to its function as a key player in the oxidative stress response, and tumourigenesis, including MM.^34^, ^35^, ^36^, ^37^ To show that NRF2 is targeted by miR‐224 in myeloma cells, we transfected KMS27 and KHM1B with miR‐224 mimic or NC and examined the effect of miR‐224 on NRF2 expression. Overexpression of miR‐224 reduced NRF2 protein expression in KMS27 and KHM1B cells (Figure 4B), while inhibition of endogenous miR‐224 significantly increased NRF2 protein level in DELTA47 cells (Figure 4B). NRF2 mRNA expression was also reduced in KMS27, KHM1B, and KO‐TAZ DELTA47 cells after miR‐224 mimic transfection, whereas transfection of miR‐224 inhibitor in DELTA47, DELTA47‐pLENTI, and TAZ‐KMS27 cells increased the mRNA expression of NRF2 (Figure 4C). These findings suggest that NRF2 is possibly downregulated by miR‐224 in MM.

### 3.5 TAZ is involved in miR‐224‐induced repression of NRF2

To confirm that TAZ is involved in miR‐224‐mediated repression of NRF2, first, we examined the consequence of altering TAZ expression on NRF2 levels in DELTA47 cells. TAZ knockout in DELTA47 cells significantly decreased the expression of miR‐224 which is in keeping with our initial findings (Figure 2). However, the expressions of NRF2 mRNA and some of its selected targets were increased in KO‐TAZ compared with the DELTA47‐pLENTI cells (Figure 4D). Specifically, NRF2 was increased by 2.65 ± 0.55 in KO‐TAZ DELTA47 cells compared to pLENTI cells (Figure 4D). NRF2 targets,^38^, ^39^ including HMOX1, TXR, and TXNRD1 were increased 2.15 ± 0.22‐fold, 5.52 ± 0.24‐fold, and 42.35 ± 3.36‐fold, respectively in KO‐TAZ ‐DELTA47 cells (Figure 4D). Similarly, overexpression of TAZ in KMS27 increased the expression of miR‐224 as earlier noted (Figure 2D), whereas the expression of NRF2 and some of its transcriptional targets were reduced (Figure 4E). Probing publicly available datasets, TAZ was observed to have an inverse relationship with NRF2 expression (Figure 4F). These results not only identify NRF2 as a miR‐224 target that regulates response to oxidative stress, but also validate the ability of TAZ to influence NRF2 and its antioxidant network through miR‐224.

KEAP1 post‐transcriptionally regulates NRF2 through a process of ubiquitylation and degradation.^24^, ^25^, ^26^ Therefore, we further examined whether TAZ modulates KEAP1 to regulate NRF2. TAZ knockout in DELTA47 cells does not affect the expression of KEAP1 (Supplementary Figure 5).

### 3.6 miR‐224 overexpression promotes susceptibility of HMCLs to frontline antimyeloma therapies

**FIGURE 5:**
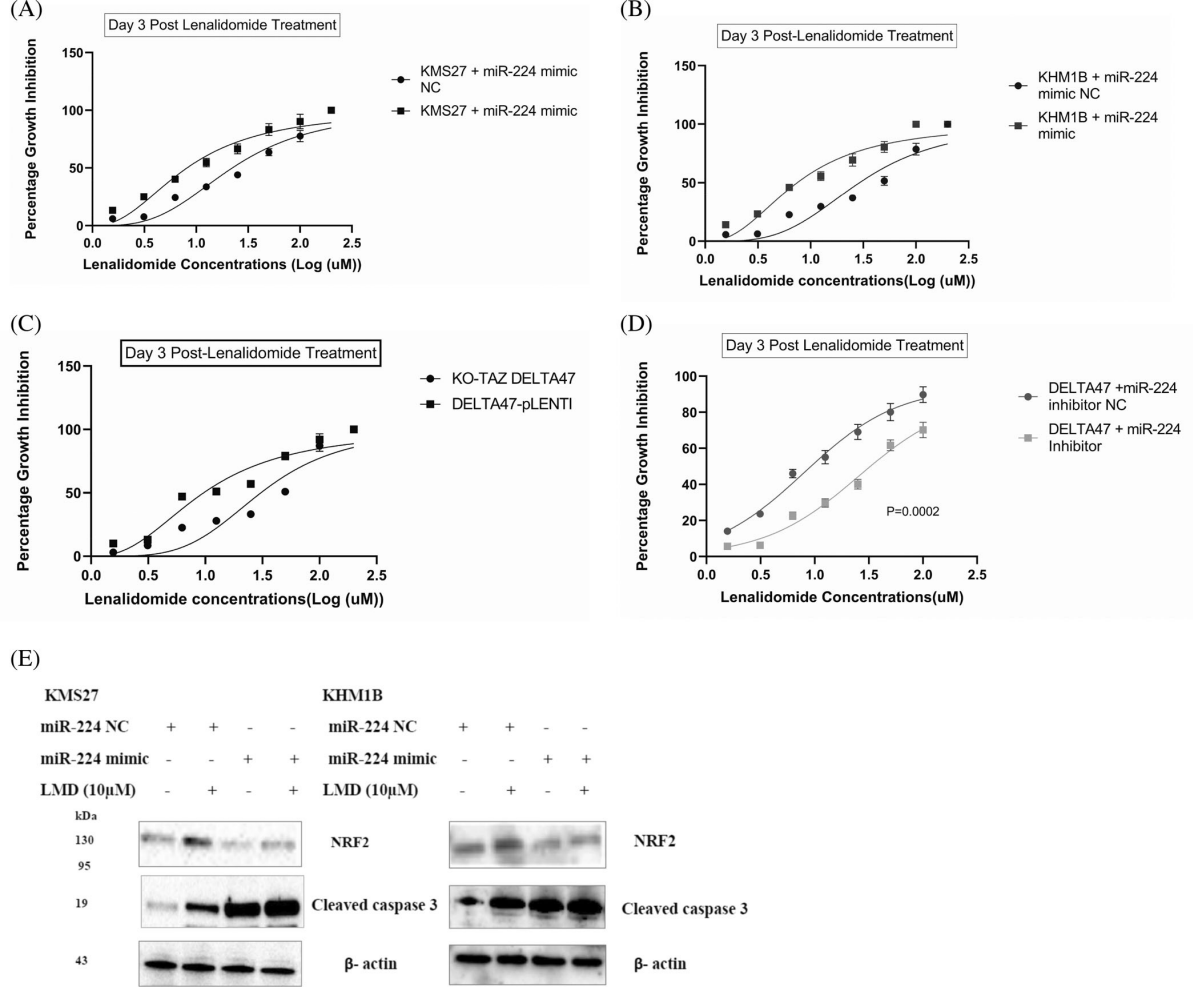
Overexpression of miR‐224 increases HMCLs' sensitivity to lenalidomide. Lenalidomide growth inhibition assay using Presto Blue stain. (A) KMS27 and (B) KHM1B cells, both transfected with miR‐224 mimic or negative control, (C) DELTA47‐PLENTI and KO‐TAZ DELTA47 (D) DELTA47‐PLENTI cells transfected with miR‐224 inhibitor or inhibitor‐negative control were treated at equal densities in 96‐well plates with LMD for 72 h. Data represent the mean ± SD from triplicate experiments. *p < .05 and **p < .0001 using the ANOVA test. (E) Immunoblot analysis of NRF2 expression in KMS27 and KHM1B cells transfected with miR‐224 mimic or negative control followed by treatment with 10 μM Lenalidomide for 72 h. Relative expression was normalized to β‐actin.

Ablation of NRF2 has been shown to restore myeloma cells' sensitivity to antimyeloma therapeutics.^36^, ^37^ Lenalidomide is a classical immunomodulatory drug (IMiD) used in treating MM.^40^ A recent study^41^ suggests that myeloma cells with less capacity to handle oxidative stress are more sensitive to lenalidomide. Because miR‐224 repressed NRF2 expression and increased HMCL vulnerability to oxidative stress (Figure 3C), we postulated that miR‐224 overexpression may influence MM sensitivity to frontline antimyeloma therapies. In both KMS27 and KHM1B cells, overexpression of miR‐224 significantly decreased the IC50 values of lenalidomide compared to negative controls (*p* < .0001). KMS27 and KHM1B cells overexpressing miR‐224 have IC50 values of 10.25 ± 0.29 and 9.49 ± 0.27 μM, respectively, whereas their negative controls have IC50 of 29.36 ± 4.47 and 32.02 ± 1.55 μM, respectively, suggesting an increased sensitivity to lenalidomide. (Figure 5A,B). To confirm that these observations are associated with TAZ, we tested the sensitivity of KO‐TAZ DELTA47 and DELTA47‐pLENTI cells to lenalidomide with a growth inhibition assay. KO‐TAZ DELTA47 cells showed lower percentage growth inhibition compared to DELTA47‐pLENTI cells (Figure 5C). Specifically, TAZ knockout in DELTA47 cells increased their IC50 value to lenalidomide from 12.25 ± 0.57 to 33.54 ± 1.63 μM suggesting that TAZ deficiency reduces myeloma cell sensitivity to lenalidomide. In contrast, DELTA47 cells transfected with miR‐224 inhibitor displayed increased resistance to lenalidomide growth inhibition, whereas miR‐224 overexpression in KO‐TAZ DELTA47 strongly enhanced the sensitivity of the cells to lenalidomide (Figure 5D). We observed that the enhanced susceptibility to lenalidomide corresponds with reduced NRF2 protein expression in the myeloma cells (Figure 5E). These results suggest that miR‐224 may mediate how myeloma cells respond to lenalidomide.

BTZ is a proteasomal inhibitor used for treating newly diagnosed or refractory MM patients.^42^ Like lenalidomide, similar results were observed when miR‐224 was overexpressed in KMS27 and KHM1B cells before treatment with BTZ (Supplementary Figure 3A–C). However, overexpression of miR‐224 inhibitor in DELTA47 cells before treatment with BTZ enhanced their resistance to BTZ was reduced (Supplementary Figure 3D). Overall, these findings suggest that miR‐224 upregulation may be a new molecular strategy to counter the resistance of myeloma cells to existing frontline antimyeloma agents.

## 4 DISCUSSION

The HP modulates cellular response to physiological and pathological stresses via YAP/TAZ.^8^, ^9^, ^10^, ^11^ We used HMCLs and MM patient samples to demonstrate that TAZ modulates the oxidative stress response of MM. We provide evidence that TAZ increases MM sensitivity to oxidative stress by repressing antioxidant mechanisms in MM cell‐line models (Figure 1A–F). We show that TAZ‐deficient myeloma cells exhibit increased resistant to oxidative stress and anti‐myeloma therapeutics (Figures 1C and 5C). Further support of the in‐vitro and in‐vivo relevance of miR‐224 in repressing antioxidant capacity in myeloma cells will require future experiments using ROS scavengers such as catalase and superoxide dismutase in addition to glutathione assays in HMCLs and MM mouse models, with genetic manipulation of miR‐224 expression or activity, and these studies are planned. Our observations suggest that TAZ suppresses myeloma cells' ability to withstand oxidative stress via the upregulation of miR‐224 expression to downregulate the NRF2 transcriptional program.

Our results showed that loss of TAZ leads to increased ROS levels in myeloma cells (Figure 1F). A possible explanation for this finding is that ROS may be acting as tumor‐promoting signaling molecules in myeloma cells. In line with these findings, previous studies^11^, ^38^ suggest cancer cells may produce a high amount of ROS to activate intracellular tumor‐promoting signals. However, excessive ROS formation can promote oxidative stress and trigger cancer cell death. Therefore, cancer cells may produce a high amount of antioxidant molecules to mop up excess ROS and maintain a ROS level that activates tumor‐promoting signals without triggering cell death.

Several reports have linked miRNAs with MM tumourigenesis and resistance to therapeutic drugs.^43^, ^44^, ^45^ However, no miRNAs have been implicated in oxidative stress phenotype in MM. Herein, we show that TAZ is associated with distinct miRNA signatures, including miR‐130a, miR‐146a, and miR‐224 in MM. Our results suggest that miR‐224 acts downstream of TAZ to suppress both tumor growth and oxidative stress resistance in MM (Figures 2 and 3). To validate these findings, we provide evidence that genetic overexpression of miR‐224 induces cell death response (Figure 3A–C) and increases myeloma cells' susceptibility to oxidative injury (Figure 3D–I). Importantly, miR‐224 overexpression sensitizes HCMLs to antimyeloma therapeutics, whereas inhibition of miR‐224 enhances myeloma resistance to targeted chemotherapies (Figure 5A,B,E, and Supplementary Figure 4A–D). In contrast to our reports, Ma et al.^46^, ^47^ demonstrate that TAZ enhances tumor growth in osteosarcoma by inducing miR‐224 to downregulate SMAD4, suggesting an oncogenic role for TAZ and miR‐224. These observations highlight the cell context‐dependent nature of TAZ and miR‐224 functions in cancer. Because miR‐224 affects cell proliferation in myeloma cells, it is possible that other miR‐224 targets other than NRF2, such as SMADs^46^, ^47^ may be involved in myeloma cell proliferation.

Some reports showed that YAP/TAZ acts non‐canonically independent of a TEAD‐mediated mechanism to induce cell death in MM and other hematological cancer.^12^, ^13^ Other contrasting studies suggest that YAP/TAZ may act canonically or through TEAD‐dependent mechanisms to regulate their targets and cancer cell growth.^48^, ^49^, ^50^ Our current observation further suggests that TAZ acts through a TEAD‐independent mechanism to induce miR224 expression in MM.

MiR‐224 is downregulated in prostate,^51^ lung,^52^ ovarian cancer,^53^ whereas it is upregulated in medulloblastoma,^54^ liver,^55^ and renal cancer,^33^ suggesting a potential context or cell type‐specific expression of miR‐224. Few studies have reported that miR‐224 correlates with the expression of GABRE in cancer.^51^, ^56^ miR‐224 is an intronic miRNA located on the GABRE gene,^52^, ^53^, ^54^ however, our findings suggest that TAZ regulation of miR‐224 in myeloma cells occurs independent of GABRE. Therefore, miR‐224 may have an independent promoter that is activated by TAZ via a TEAD‐independent mechanism in MM. The exact mechanisms through which TAZ upregulate miR‐224 remain an important area for future study.

Furthermore, we show that miR‐224 represses NRF2 to mediate myeloma cell response to oxidative stress and antimyeloma agents (Figure 4A–F). Although the 3′UTR of NRF2 contains miR‐224 binding sites, other indirect mechanisms may be involved in repressing NRF2. We did not perform any experiments to test the notion that mutating the recognition sequence in the NRF2 3′UTR will render it insensitive to miRNA‐224 and thus protects cells from oxidative stress. Therefore, future studies using reporter assays are needed to test this hypothesis. Several studies suggest that NRF2 mediates tumor growth, metabolism, and chemotherapy resistance in solid tumors.^38^, ^39^, ^57^, ^58^ In agreement with these reports, previous studies in leukemia, lymphomas, and MM have shown that upregulation of the NRF2 antioxidant pathway increases tumor resistance to cytotoxic chemotherapy.^59^, ^60^, ^61^, ^62^ Herein we show that miR‐224 or TAZ may downregulate NRF2 expression in myeloma cells (Figure 4B–D) to enhance their sensitivity to antimyeloma chemotherapies. Importantly, NRF2 expression in patient samples shows an inverse relationship with TAZ in a publicly available database (Figure 4F). TAZ or miR224 transcriptional regulation of NRF2 expression uncovers the scientific justification to combine frontline therapies such as IMiDs and BTZ with miR‐224 mimic therapy or anti‐NRF2 therapies such as halofuginone and luteolin (3′,4′,5,7‐tetrahydroxyflavone).^63^, ^64^ Halofuginone decreases NRF2 protein synthesis in NRF2‐addicted cancer cells by inhibiting prolyl‐tRNA synthetase,^63^ while luteolin inhibits mRNA and protein expression of NRF2 in A549 cells.^64^, ^65^

HMOX1 is a key survival molecule controlled by NRF2 and involved in the progression of tumors in many cancers^66^, ^67^ We showed that the levels of HMOX1 and some other known targets of NRF2 are upregulated in our TAZ knockout MM model and negatively correlated with TAZ (Figure 4D,E). These observations further confirm the control of the NRF2 transcriptional program by TAZ through miR‐224. To our knowledge, this is the first evidence to suggest that loss or low expression of TAZ enables a high amount of antioxidant ROS scavengers to be expressed in myeloma cells. Such unique feature enables tumor promoting signals to be activated without triggering cancer cell death.

Although, we did not investigate the effect of miR‐130a and miR‐146a on oxidative stress response in MM, nevertheless, our findings provide a rationale for future study to evaluate the clinical relevance of miR‐130a and miR‐146a on antioxidant mechanisms in MM.

In terms of study limitations, glutathione assay was the only method of quantitation used to assess cellular antioxidant response in this study. Therefore, future studies should consider using more than one analytic method, for example, total oxygen radical absorbance capacity (ORAC) and glutathione assays. The protein expression findings in the current study were based on a subjective interpretation of immunoblot data, however we plan to adopt more objective quantitative image analysis in the next stage of this work that involves preclinical evaluation of the therapeutic effect of miR‐224 mimic on MM mouse models.

In conclusion, we have shown that TAZ inhibits cellular response to oxidative stress by upregulating miR‐224 to post‐transcriptionally repress NRF2 antioxidant network and promote cell death in MM. We further confirmed that upregulating miR‐224 in myeloma cells may increase sensitivity to antimyeloma therapies. Importantly, the repression of NRF2 by TAZ or miR‐224 in MM oxidative stress response may have a critical impact on studies in antimyeloma therapies.

## AUTHOR CONTRIBUTIONS

**Tony Reiman:** Funding acquisition (lead); resources (equal); supervision (lead); writing – review and editing (lead). **Samuel O. Abegunde:** Conceptualization (lead); data curation (lead); formal analysis (lead); investigation (lead); methodology (lead); project administration (lead); resources (equal); software (equal); validation (equal); visualization (equal); writing – original draft (lead). **Stacy Grieve:** Conceptualization (supporting); data curation (supporting); investigation (supporting); methodology (supporting); validation (equal); writing – review and editing (equal).

## FUNDING INFORMATION

We wish to acknowledge the Canadian Institutes of Health Research, Terry Fox Research Institute and New Brunswick Innovation Foundation for their funding support.

## CONFLICT OF INTEREST STATEMENT

The authors have stated explicitly that there are no conflicts of interest in connection with this article.

## ETHICS STATEMENT

Approval of the research protocol by an Institutional Reviewer Board: N/A. Informed Consent: N/A. Registry and the Registration No. of the study/trial: N/A. Animal Studies: N/A.

## Supporting information


**Supplementary Figure 1** (A) Bubble assay comparing oxygen release from H_2_O_2_ in WT‐TAZ DELTA47 (DELTA47‐pLENTI) and KO‐TAZ DELTA47 cells.
**Supplementary Figure 2** Immunoblot showing TAZ‐F52A/F53A, TAZ or WPI in KMS 27 and KHM1B cell lines. β‐actin was used as a loading control.
**Supplementary Figure 3** Overexpression of miR‐224 increases sensitivity of HMCLs to BTZ‐mediated cytotoxicity. Bortezomib growth inhibition assay using Presto Blue stain. Low TAZ expressing HMCLs exemplified by (A) KMS27 and (B) KHM1B cells transfected with miR‐224 mimic or negative control were treated with increasing concentrations of BTZ for 48 h. Data represents the mean ± SD from triplicate experiments. *p < .05 and **p < .0001 using the ANOVA test. Immunoblot analysis showing NRF2 and cleaved caspase3 expression in (C) KMS27 transfected with miR‐224 mimic or negative control followed by treatment with BTZ for 48 h. (D) Bortezomib growth inhibition assay in DELTA47 cells transfected with miR‐224 inhibitor or negative control and treated with BTZ for 48 h.
**Supplementary Figure 4** Genetic inhibition of GABRE in DELTA47 cells has no significant effect on miR‐224 expression. miR‐224 expression in DELTA47 cells 48 h after 48 h post siGABRE transfection. Data are mean ± SD of triplicates.
**Supplementary Figure 5** Relative expression of KEAP1 mRNA in DELTA47pLENTI versus KO‐TAZ DELTA47 myeloma cells. Data are mean ± SD of triplicates. ns, not significant.

**Supplementary Table 1** Quantitative PCR primers.

**Data S1** Supporting information.

## Data Availability

The data that support the findings of this study are available from the corresponding author upon reasonable request.
