# Supplementary material for: TAZ upregulates MIR‐224 to inhibit oxidative stress response in multiple myeloma
Source: Cancer Rep (Hoboken). 2023 Aug 4;6(10):e1879. doi: 10.1002/cnr2.1879 (PMC10598259; doi:10.1002/cnr2.1879)

**Supplementary Files**

**Supplementary Materials and Methods:**

**Transient knockdown of GABRE with siRNA**

GABRE was knocked down using silencer™ pre-designed siRNA (Ambion, USA) in according to the manufacturer’s instruction, with universal scrambled negative siRNA as control. We assessed the efficiency the knockout 48 hours after transfection using Western blot analysis.

**Supplementary Results:**

**Supplementary Figure 1**


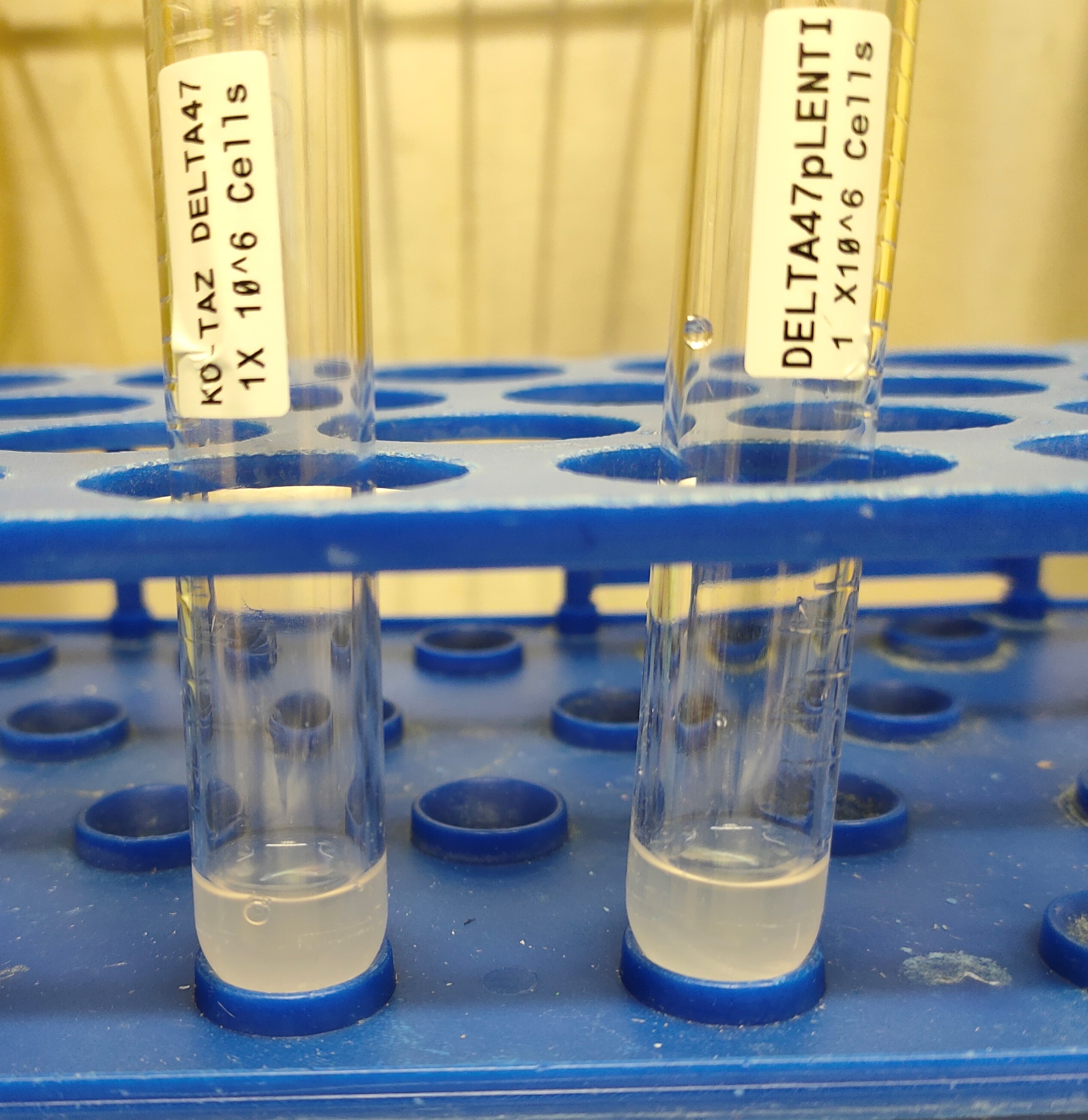


**Supplementary Figure 2**

**Supplementary Figure 3**

**A**


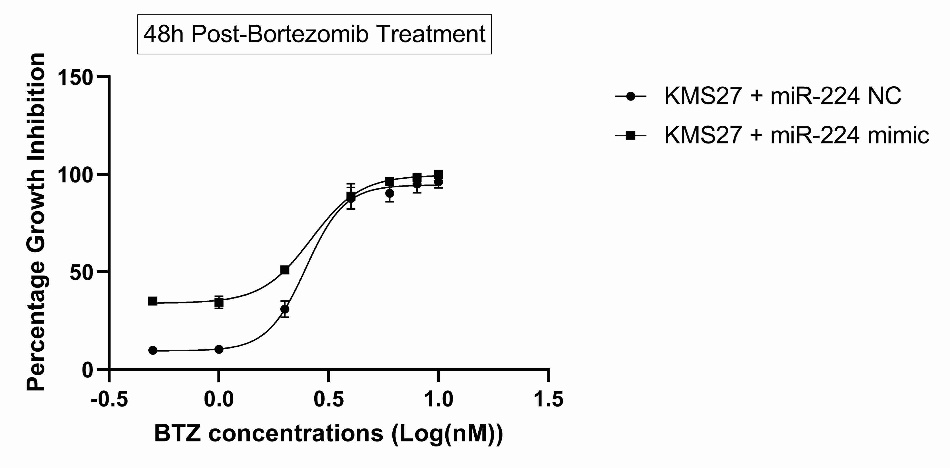


**B**


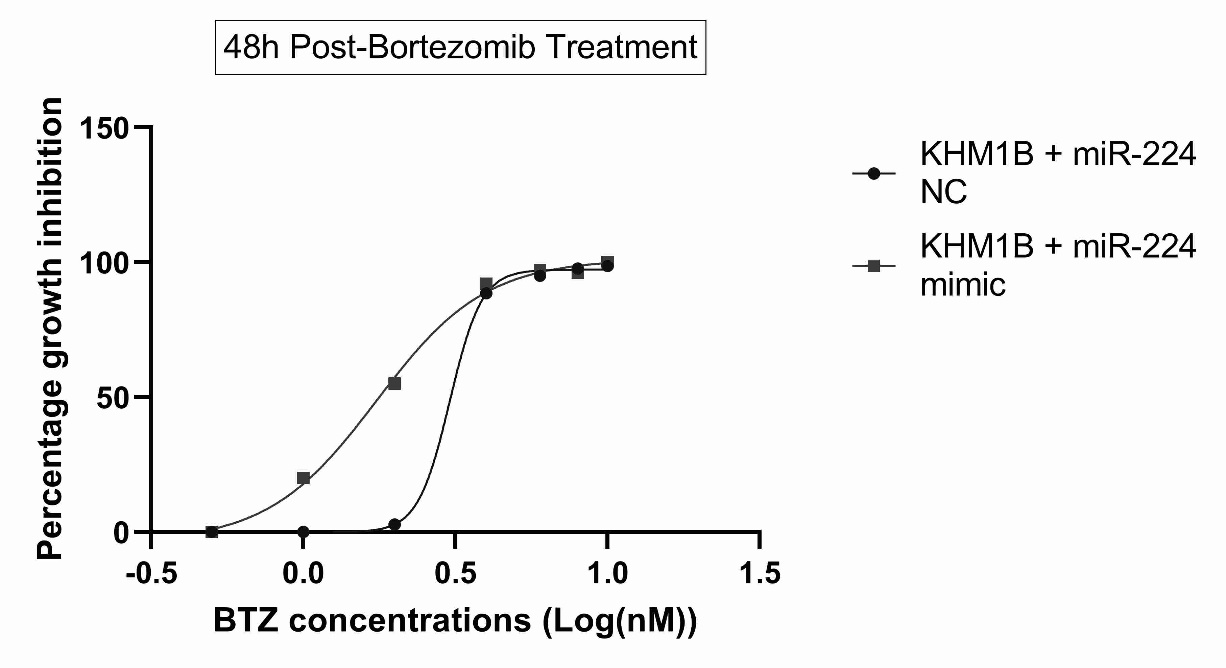


**C**

**D**


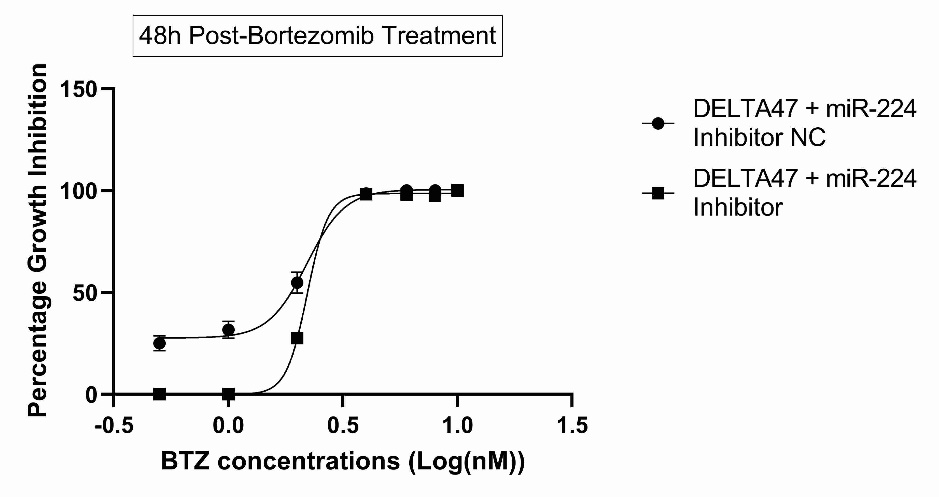


**Supplementary Figure 4**


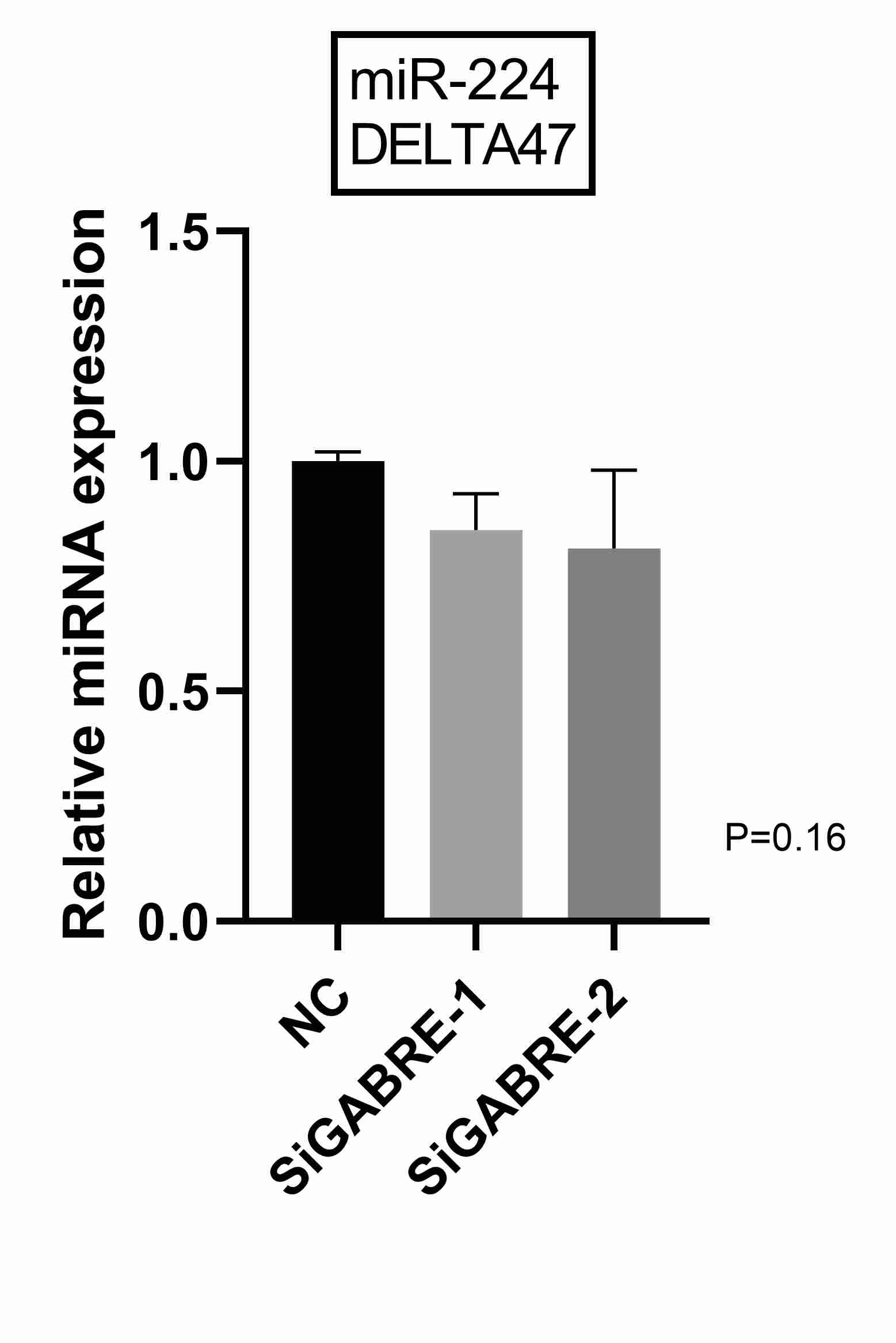


**Supplementary Figure 5**


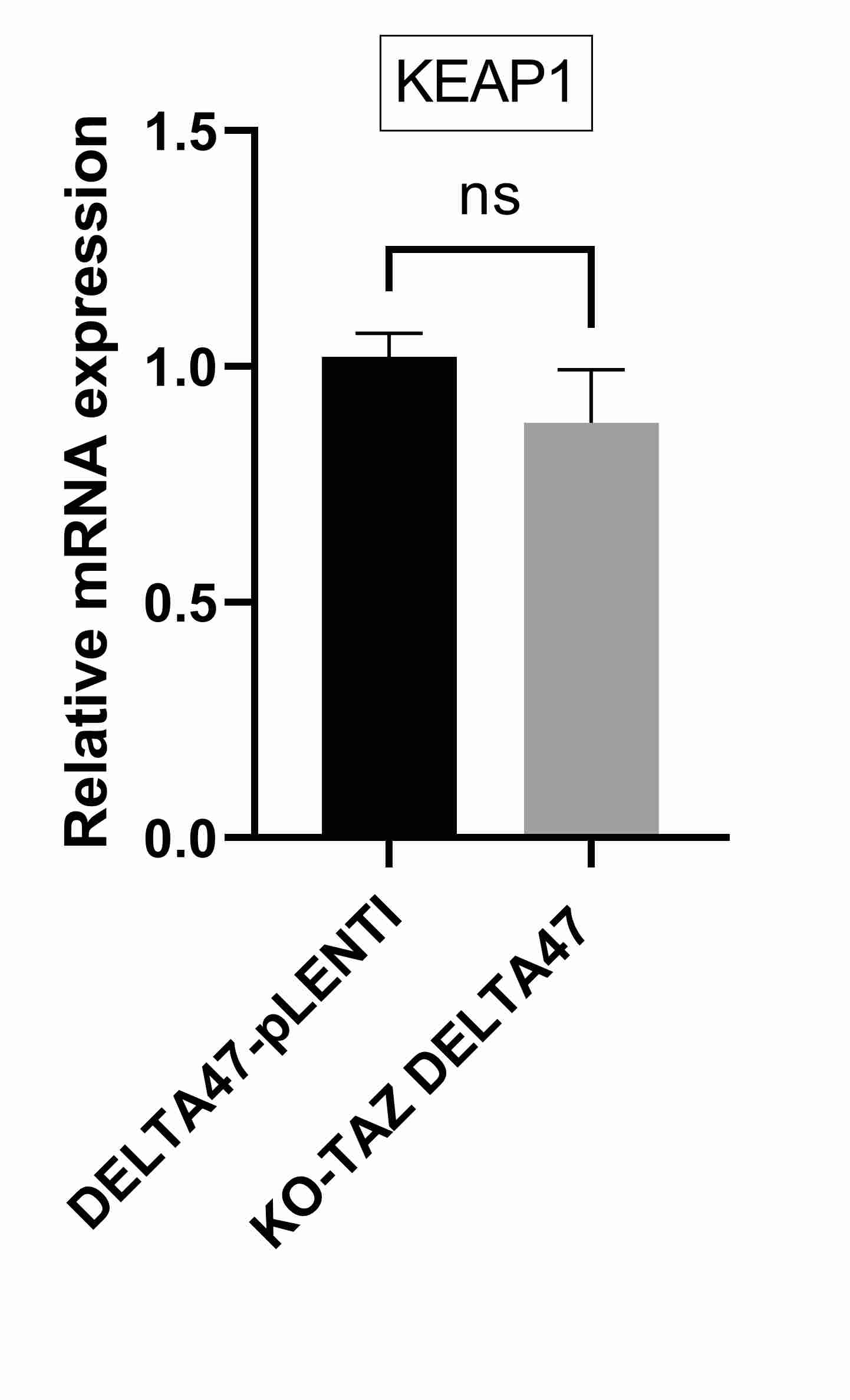

Supplement: Supplementary file 1 — Supplementary Figure 1 (A) Bubble assay comparing oxygen release from H2O2 in WT‐TAZ DELTA47 (DELTA47‐pLENTI) and KO‐TAZ DELTA47 cells. Supplementary Figure 2 Immunoblot showing TAZ‐F52A/F53A, TAZ or WPI in KMS 27 and KHM1B cell lines. β‐actin was used as a loading control. Supplementary Figure 3 Overexpression of miR‐224 increases sensitivity of HMCLs to BTZ‐mediated cytotoxicity. Bortezomib growth inhibition assay using Presto Blue stain. Low TAZ expressing HMCLs exemplified by (A) KMS27 and (B) KHM1B cells transfected with miR‐224 mimic or negative control were treated with increasing concentrations of BTZ for 48 h. Data represents the mean ± SD from triplicate experiments. *p < .05 and **p < .0001 using the ANOVA test. Immunoblot analysis showing NRF2 and cleaved caspase3 expression in (C) KMS27 transfected with miR‐224 mimic or negative control followed by treatment with BTZ for 48 h. (D) Bortezomib growth inhibition assay in DELTA47 cells transfected with miR‐224 inhibitor or negative control and treated with BTZ for 48 h. Supplementary Figure 4 Genetic inhibition of GABRE in DELTA47 cells has no significant effect on miR‐224 expression. miR‐224 expression in DELTA47 cells 48 h after 48 h post siGABRE transfection. Data are mean ± SD of triplicates. Supplementary Figure 5 Relative expression of KEAP1 mRNA in DELTA47pLENTI versus KO‐TAZ DELTA47 myeloma cells. Data are mean ± SD of triplicates. ns, not significant. [file CNR2-6-e1879-s002.docx]
