# Supplementary material for: TAZ upregulates MIR‐224 to inhibit oxidative stress response in multiple myeloma
Source: Cancer Rep (Hoboken). 2023 Aug 4;6(10):e1879. doi: 10.1002/cnr2.1879 (PMC10598259; doi:10.1002/cnr2.1879)
Supplement: Supplementary file 2 — Supplementary Table 1 Quantitative PCR primers. [file CNR2-6-e1879-s003.docx]

**Supplementary Table1: Quantitative PCR Primers**

| **No** | **Oligo Name** | **Nucleotide sequence (5’-3’)** |
| --- | --- | --- |
| 1 | qrRNA-F | TCCCCATGAACGAGGAATTCC |
| 2 | qrRNA-R | AACCATCCAATCGGTAGTAGC |
| 3 | qTAZ-F | GTATCCCAGCCAAATCTCGTGATG |
| 4 | qTAZ-R | CACCGCATTGGGCATACTCATG |
| 5 | qNRF2-F | CACATCCAGTCAGAAACCAGTGG |
| 6 | qNRF2-R | GGAATGTCTGCGCCAAAAGCTG |
| 7 | qCYR61-F | GAAGCGGCTCCCTGTTTTTG |
| 8 | qCYR61-R | CGGGTTTCTTTCACAAGGCG |
| 9 | qCTFG-F | TGGAGATTTTGGGAGTACGG |
| 10 | qCTFG-R | CAGGCTAGAGAAGCAGAGCC |
| 11 | qThioredoxin-F | TGAAGCAGATCGAGAGCAAGAC |
| 12 | qThioredoxin-R | TTCATTAATGGRGGCRRCAAGC |
| 13 | qThioredoxin Reductase-F | GAAGATCTTCCCAAGTCCTATGAC |
| 14 | qThioredoxin Reductase-R | ATTTGTTGCCTTAATCCTGTGAGG |
| 15 | qHMOX1-F | CCAGGCAGAGAATGCTGAGTTC |
| 16 | qHMOX1-R | AAGACTGGGCTCTCCTTGTTGC |
| 17 | U6 -F | GTGCTCGCTTCGGCAGCACATATAC |
| 18 | U6-R | AAAAATATGGAACGCTTCACGAATTTG |
| 19 | miR-224-F | CAAGTCACTAGTGGTTCC |
| 20 | miR-224-R | GAACATGTCTGCGTATCTC |
| 21 | miR-146a-F | GAGAACTGAATTCCATGG |
| 22 | miR-146a-R | GAACATGTCTGCGTATCTC |
| 23 | miR-130A-F | CACATTGTGCTACTGTCT |
| 24 | miR-130A-R | GAACATGTCTGCGTATCTC |
| 25 | KEAP1-F | CAACTTCGCTGAGCAGATTGGC |
| 26 | KEAP1-R | TGATGAGGGTCACCAGTTGGCA |
| 27 | GABRE-F | TGGCAAACTGCCAGAAGCCTCT |
| 28 | GABRE-R | ACCAAGGCTGTTGACGGAGATC |
