# Supplementary material for: TAZ upregulates MIR‐224 to inhibit oxidative stress response in multiple myeloma
Source: Cancer Rep (Hoboken). 2023 Aug 4;6(10):e1879. doi: 10.1002/cnr2.1879 (PMC10598259; doi:10.1002/cnr2.1879)
Supplement: Supplementary file 3 — Data S1 Supporting information. [file CNR2-6-e1879-s001.pdf]

1. **Raw Western Blot Image containing a portion of data used for Figure 1A.**

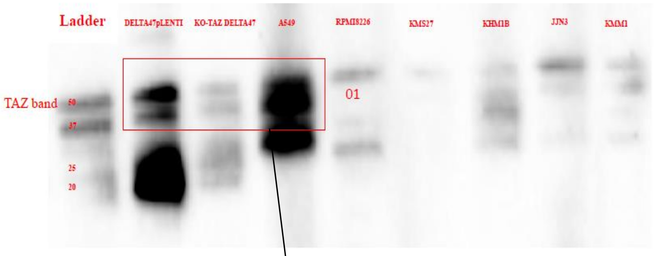

Figure 1A

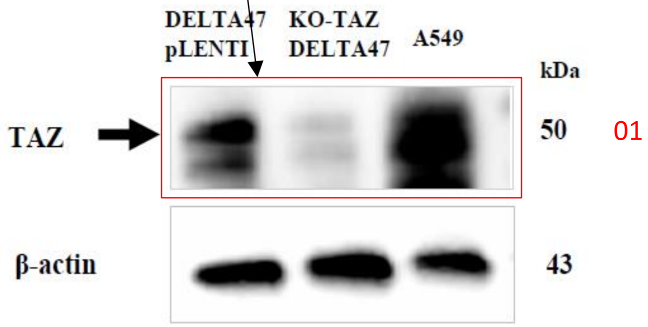

2. **Raw Western Blot Image containing a portion of data used for Figure 3C.**

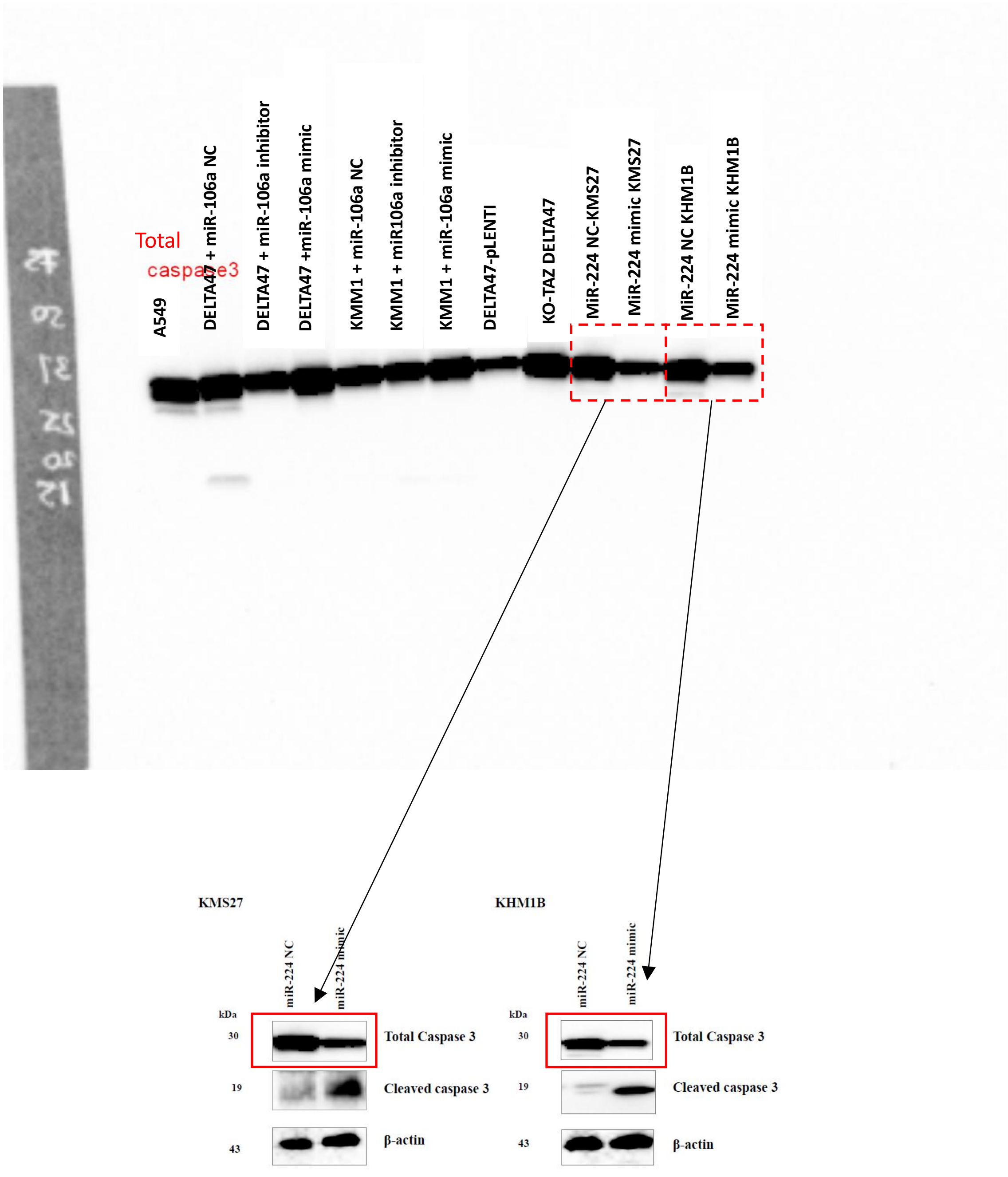

Expanded data with ladder marker to support part of Fig 3C data: Total Caspase3 protein expression in selected HMCLs, DELTA47pLENTI, KO-TAZ DELTA47, miR-224NC-KMS27, miR-224 mimic KMS27 cells, miR-224 NC KHM1B, and miR-224 mimic KHM1B relative to A549 cells.

3. **Raw Western Blot Image containing data used for Supplementary figure 3C.**

KMS27

**miR 224 mimic**	+	+	+	-	-	-
**miR 224 NC**	-	-	-	+	+	+
**2 nM BTZ**	-	+	-	-	+	-
**4 nM BTZ**	-	-	+	-	-	+

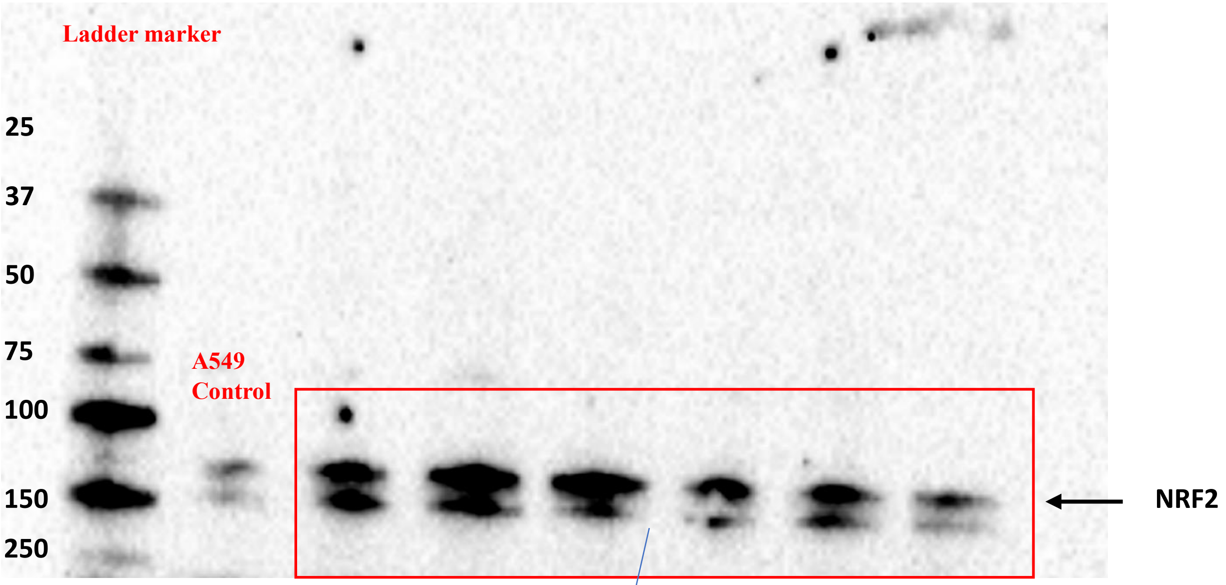

Supplementary Figure 3C

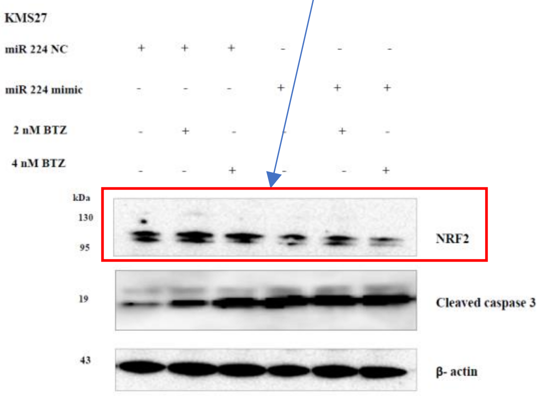

Expanded data with ladder marker of Supplementary Fig 3C data: Immunoblot analysis showing NRF2 expression in (C) KMS27 cells transfected with miR-224 mimic or negative control followed by treatment with BTZ for 48 hours. A549 was used as control.
